# Different neurocognitive patterns of conflict control in Tibetans living above and below 4,000 m

**DOI:** 10.7717/peerj.7269

**Published:** 2019-07-08

**Authors:** Hailin Ma, Buxin Han, Yan Wang

**Affiliations:** 1 Center on Aging Psychology, CAS Key Laboratory of Mental Health, Institute of Psychology, Beijing, China; 2 University of Chinese Academy of Sciences, Beijing, China; 3 Plateau Brain Science Research Center, Tibet University/South China Normal University, Guangzhou/Tibet, China

**Keywords:** Altitude threshold, Conflict control, Event-related potentials (ERPs), Flanker task, Tibetan

## Abstract

**Background:**

The existence of a particular threshold of hypoxia severity, beyond which neuropsychological functioning is compromised, is unclear. We investigated the neurocognitive profile related to conflict control in healthy young Tibetans born and living at three different altitudes (2,700 m, 3,700 m, and 4,500 m) in Tibet to investigate the existence of this threshold.

**Methods:**

Using event-related potentials (ERPs), the conflict control functions of individuals in the three altitude groups were investigated by means of a flanker task, using congruent and incongruent stimuli. The data were analyzed using mixed-model analyses of variance.

**Results:**

Although effect of altitude was not significant at a behavioral level (*p* > 0.05), the ERPs showed cognitive conflict modulation. The N2 difference wave (for incongruent minus congruent conditions) was smaller in the 4,500-m group than in the groups living below 4,000 m (*p* < 0.05). The study’s findings suggest that the influence of high altitude in the conflict monitoring stage becomes significant above 4,000 m. Thus, the altitude threshold for impairment of cognition may be 4,000 m.

## Introduction

According to the sixth national population census in China conducted in 2010 (China, 2012), more than 6.5 million Tibetans permanently live on the Qinghai–Tibetan Plateau. Approximately 14.89% of these individuals live at altitudes between 2,500 and 3,000 m, 75.05% live at altitudes between 3,000 and 4,000 m, and 10.06% live at altitudes above 4,000 m. The amount of oxygen in the air is reduced at high altitudes, resulting in lower arterial oxygen saturation (SpO_2_), that is, a state of hypoxia, which is known to affect cognition (Virués-Ortega et al., 2011).

Long-term inhabitation at high altitudes, may lead to impaired conflict control processing, due to hypoxia. The brain regions which are involved in conflict control, were found to be affected by long-term exposure to high altitude. The prefrontal cortex showed decreased gray matter volume, bilaterally, in individuals living at high altitudes (Yan et al., 2010). Moreover, cerebral blood flow was decreased in the right parietal lobe and the anterior cingulate cortex (ACC) (Wang et al., 2018). In our earlier event-related potential (ERP) study, the high-altitude group (people born at low altitude, but who had lived at high altitudes for 3 years) demonstrated delayed NoGo-N2 latency compared with the low-altitude group in the executive control test, which suggested that the processing speed during the conflict monitoring stage was influenced by long-term high-altitude exposure (Ma et al., 2015a). Moreover, we compared the cognitive differences among the Han ethnic groups living at high and low altitudes by using a flanker task to assess their conflict control function. During the incongruent trial, a smaller P3 amplitude was found in the high-altitude group than in the low-altitude group, which suggests that the conflict-resolving stage of conflict control processing was influenced by long-term exposure to high altitudes (Ma et al., 2015b). However, the influence of high altitude on conflict control processing among individuals indigenous to these regions remains unclear.

In areas of high-altitude, cognitive impairment varies with altitude, and a conflict control-impairment threshold may exist at 4,000 m. The first evidence of this hypothesis was observed in a physiological study, which showed that individuals living at altitudes higher than 4,000 m demonstrate loss of cerebral auto-regulation (Jansen et al., 2007), suggesting the potential influence of hypoxia, which may compromise neuropsychological function (Virués-Ortega et al., 2011). In another behavioral study (4-choice reaction-time test), reaction time was significantly impaired at altitudes above 4,000 m, but relatively unaffected below that height (Dykiert et al., 2010). Moreover, individuals inhabiting extremely high altitudes (over 4,000 m), showed lower levels of performance in all executive tests (Virués-Ortega et al., 2011). However, in contrast, three different electrophysiological studies (Ma et al., 2015a; Richardson et al., 2011; Singh et al., 2004), reported that the influence of high altitude on cognition was present at altitudes above and below 4,000 m. Our previous study (Zhang et al., 2018), based on the attention network test (ANT), compared individuals residing at 2,900 m and 3,700 m with those residing at 4,200 m. The latter had lower orienting and executive function scores. They had larger N1 and P3 amplitudes than individuals in the other groups, which suggested that high-altitude could affect attentional function in individuals indigenous to different high-altitude areas and that a threshold may exist at 4,000 m (Zhang et al., 2018). However, there has been no electrophysiological study that has compared conflict control directly between the groups residing above and below 4,000 m.

The flanker task is commonly used to measure conflict processing. Congruent and incongruent conditions are included in the task, and participants are asked to indicate the direction of a central target, while ignoring the flanker (Eriksen & Eriksen, 1974). The response time and accuracy rate in incongruent trials are generally slower and lower, respectively, than in congruent trials (Tillman & Wiens, 2011).

The high temporal resolution of ERPs enables the detection of the temporal sequence of brain processes (Luck, 2014). The cognitive processing of conflict control includes conflict detection and conflict resolution phases (Fan et al., 2003). The conflict detection or monitoring process is associated with a frontal and central N2 ERP component (Brydges et al., 2014), which occurs between 250 ms and 350 ms after stimulus onset, and peaks at *F*_Z_, FC_Z_, and C_Z_ electrode sites in the 10/20 system (Brydges et al., 2014). The N2 amplitude of the incongruent trial was higher than the congruent trial in the flanker task (Kopp, Rist & Mattler, 1996). Moreover, conflict resolution processing is associated with the P3 ERP component (Rueda et al., 2004), which is maximal at the central and parietal scalp sites (C_Z_ and CP_Z_) and occurs approximately between 300 ms and 500 ms after stimulus onset (Polich, 2007). A larger P3 amplitude was reported in the incongruent trial than in the congruent trial and was associated with careful evaluation of the stimulus to determine the correct response (Rueda et al., 2004).

In our earlier studies (Ma et al., 2015a; Ma et al., 2015b), flanker and Go/No-Go tasks were used to evaluate individuals, who were born at a low altitude and had lived at high altitudes (3,700 m) for 3 years. We found that executive control processing was influenced by high altitude. However, in high-altitude areas (>2,500 m), various altitudes influence cognitive function differently. The existence of a 4,000-m threshold for cognitive impairment and the underlying neuropsychological mechanism of the influence of various high-altitudes on conflict control are still unclear. Indigenous Tibetans living in high-altitude areas have adapted to their environment. Thus, the physiological differences between them are small, which made them suitable for inter-group comparison.

In this study, we investigated Tibetan individuals indigenous to high altitudes, to establish whether an altitude of 4,000 m was the conflict control impairment threshold, and to determine the neurocognitive profile of conflict control in different high-altitude groups. We hypothesized that conflict control processing would be highly affected in individuals residing at altitudes above 4,000 m compared to those residing at lower altitudes, in terms of reduced accuracy and increased reaction time, and that the higher-altitude group would have lower N2 and P3 amplitudes. All the participants would have faster and more accurate responses in the congruent trials relative to the incongruent trials, with higher N2 and larger and slower P3 amplitudes in the incongruent trials than in the congruent trials.

## Materials & Methods

### Participants

Fifty-two right-handed healthy young Tibetans, aged 18–24 years, participated in this study after signing informed consent forms. All of them had normal or corrected-to-normal vision. The participants were born and raised at three different altitudes (2,700 m, 3,700 m, and 4,500 m, respectively). The 2,700 m and 4,500-m groups migrated to Lhasa while they attended Tibet University, and were living at 3,700 m (Lhasa). Seventeen participants (10 men, 21.71  ± 1.10 years) lived at an altitude of 2,700 m (Nyingchi), 18 participants (8 men, 21.00  ± 1.19 years) lived at an altitude of 3,700 m (Lhasa), and 17 participants (9 men, 20.64  ± 1.46 years) lived at an altitude of 4,500 m (Nagqu). The experiment was conducted in accordance with the Declaration of Helsinki and was approved by the Ethics Committee of the Institute of Psychology, Chinese Academy of Sciences (H16037).

SpO2 and heart rate were measured on a warmed hand in the resting condition, using a pulse oximeter (CMS50D, CONTEC, China).

### Procedure

**Figure 1:**
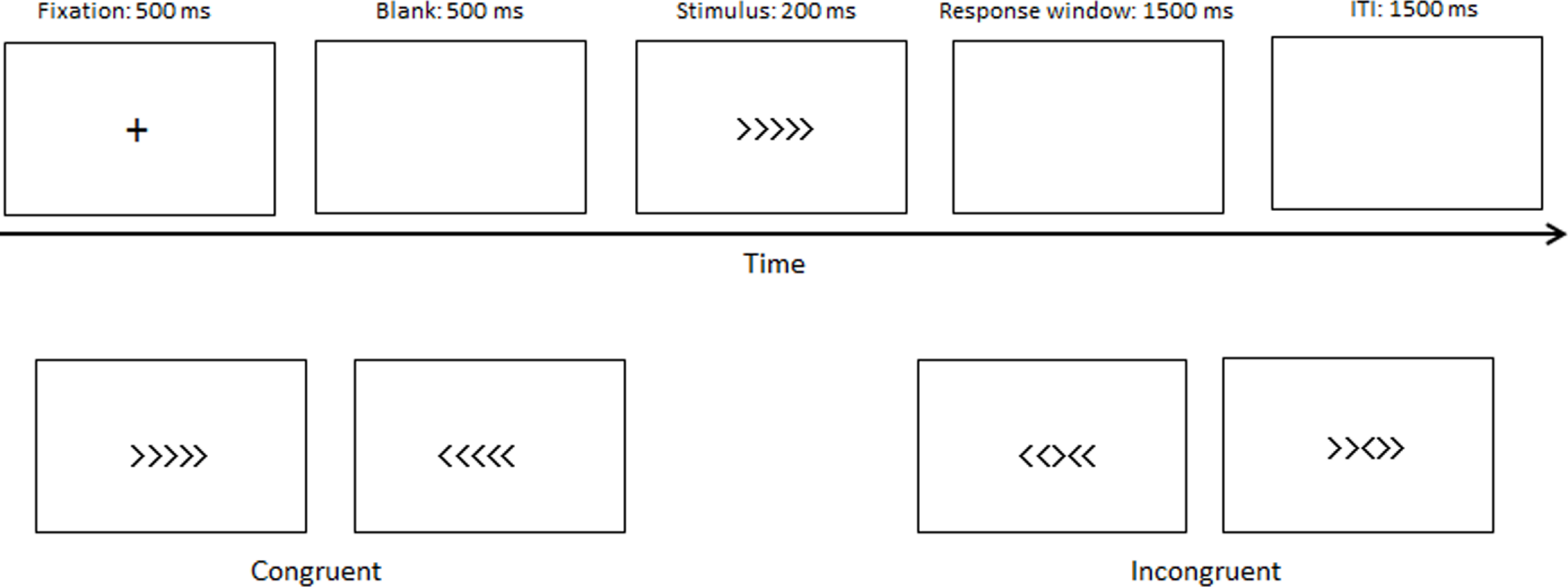
Materials and procedure. The procedure of the experimental paradigm.

An arrow version of the flanker task was used in our study. The congruent (→→ → → → or ← ← ← ← ←) and incongruent (→ →← → → or ← ← → ← ←) stimuli accounted for half, and the central arrow was the target. Participants had to perform either a left or right-hand button-press to determine the target direction. The fixation cross was presented at 500 ms, followed by a blank screen (500 ms), after which the stimuli were presented at 200 ms, followed by a 1,500 ms response window. The intertrial interval (ITI) was 1,500 ms (Fig. 1). A practice block with 24 trials was used to ensure that the participants understood the experiment. Two experimental blocks of 160 trials in total, followed the practice block, with short breaks between blocks.

### ERP Recording

Continuous electroencephalography (EEG) was performed with Ag/AgCl electrodes at 64 scalp sites, according to International 10–20 system (Neuroscan Inc., Abbotsford, VA, Australia). The physical reference electrode was placed approximately 2 cm posterior to C_Z_. Electrooculogram (EOG) data were recorded using facial electrodes, placed above and below the left eye (VEO) and at the outer canthi of both eyes (HEO). All electrode impedances were below 5 kΩ, and data were continuously recorded at a sampling rate of 500 Hz, and a filter bandwidth of 0.05–100 Hz.

Offline analysis was performed using SCAN 4.5 (Neuroscan Inc., Abbotsford, VA, Australia). The EEG data were re-referenced based on the average of the left and right mastoid (M1 and M2) electrodes. Ocular artifacts were corrected using a regression procedure with Neuroscan software (Semlitsch et al., 1986). Data were digitally filtered with a 30 Hz low-pass filter, and were divided into 1000 ms ERP segments, including a 200 ms interval before the target onset. A criterion of ±75 µV was used for artifact rejection.

Seven electrode sites (F_Z_, FC_Z_, C_Z_, F_1_, F_2_, FC_1_, and FC_2_) were selected for N2 and four electrode sites (C_Z_, CP_Z_, P_Z_, and PO_Z_) were selected for P3 data analysis, because the amplitudes of N2 and P3 were maximum at these sites. Moreover, according to earlier studies, the effect of conflict processing was obvious in these regions (Brydges et al., 2014; Tillman & Wiens, 2011). The time window for recording N2 mean amplitude was 300–420 ms. The specific time windows for ERP components were selected by visual inspection of ERP grand averages. To further analyze the negativity occurring in the incongruent condition, difference waveforms (for incongruent minus congruent conditions) of the N2 component were computed; the peak amplitude was used for statistical analysis and the electrode sites were the same as those assessing the N2 component.

The mean amplitude was also used for the P3 component, with the time windows at 340–530 ms for the congruent and at 410–600 ms for the incongruent P3 component. The 50% area latency was used for the P3 component (Di Russo et al., 2002). The sampling point where a pre-specified fraction (50% in this case) of the total area was reached was defined as the mean latency. The onset latencies of the P3 wave were determined using a combination of the jackknife method and the fractional area latency measure. In accordance with earlier studies, the statistical results (*F*-values and *t*-values) were corrected using the formulae: *FC* = *F*∕(*N* − 1)2, and *tC* = *t*∕(*N* − 1), where *N* denotes the number of observations in each condition (Ulrich & Miller, 2001).

### Data analysis

SPSS version 20 (IBM Corp., Armonk, NY, USA) for Windows was used for data analysis, and *P* < 0.05 was considered statistically significant. SpO_2_ and heart rate were compared using one-way ANOVA, with the altitude group (2,700 m, 3,700 m, and 4,500 m) as the independent variable. Mixed-model ANOVA was used to analyze the accuracy rates and the reaction times for correct responses. The independent variable was the trial type (congruent and incongruent) and was considered as the within-subject factor, and altitude group (2,700 m, 3,700 m, and 4,500 m) was treated as the between-subject factor.

A mixed two-way, repeated measure analysis of variance (ANOVA) was used to analyze the N2 and P3 components. The within-subject factors included the trial type (two levels: congruent and incongruent), and the electrode sites (seven for N2 and four for P3). The between-subject factor was the altitude group (three levels: 2,700 m, 3,700 m, and 4,500 m). One-way ANOVA was applied to the N2 difference wave, with altitude-group (three levels: 2,700 m, 3,700 m, and 4,500 m) as the between-subject factor. The Greenhouse–Geisser correction was used to compensate for sphericity violations. Simple effect analyses were conducted to explore interaction effects. Partial eta-squared was used to measure the effect size.

## Results

SpO_2_ and heart rate did not differ among the three groups (*p* = 0.28, *p* = 0.47). SpO_2_was 92.53% ± 2.32% in the 2,700-m group, 91.44% ± 2.83% in the 3,700-m group, and 91.06% ± 3.07% in the 4,500-m group. Heart rate was 75 ± 8 beats/min in the 2,700-m group, 79 ± 11 beats/min in the 3,700-m group, and 78 ± 11 beats/min in the 4,500-m group.

### Behavioral results

**Figure 2:**
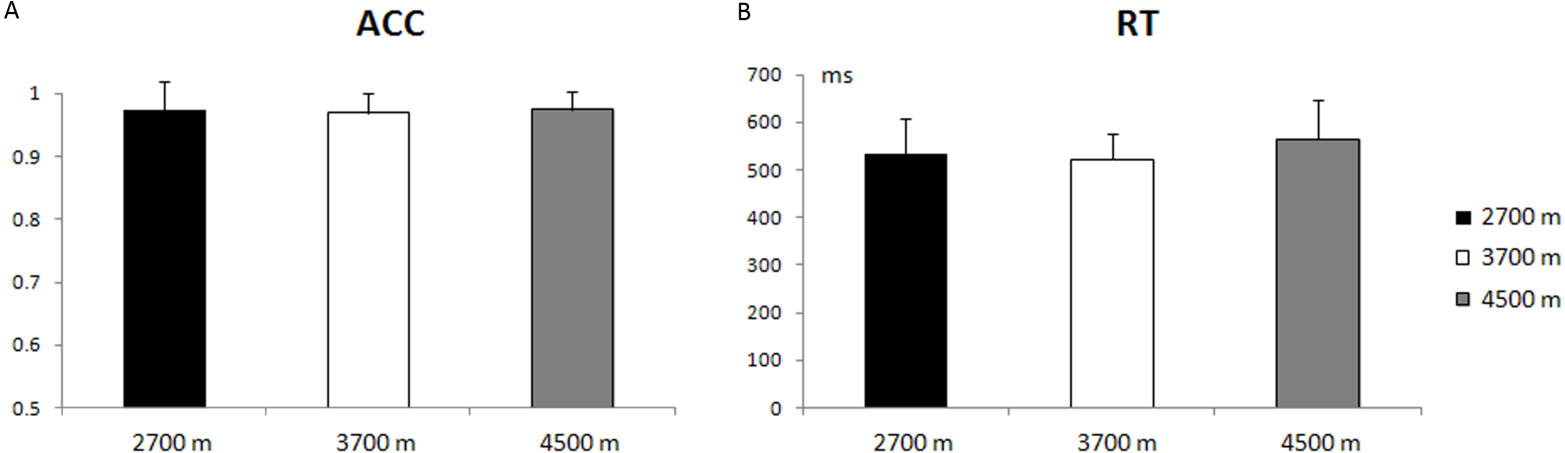
Behavioral results. (A) The mean accuracy rate (ACC) and standard error for the 2,700 m, 3,700 m, and 4,500 m groups. (B) The reaction time (RT) and standard error for the 2,700 m, 3,700 m, and 4,500 m groups.

The main effect of altitude-group was not significant, with respect to the reaction time (RT) and accuracy rate (ACC) (*p* = 0.19; *p* = 0.93). The average accuracy rate was 97.40% ± 4.45% (mean ± S.E.) for the 2,700-m group, 96.98% ± 3.10% for the 3,700-m group, and 97.39% ± 3.05% for the 4,500-m group. The average reaction time for correct responses was 533 ± 74 ms for the 2,700-m group, 522 ± 54 ms for the 3,700-m group, and 565 ± 81 ms for the 4,500-m group (Fig. 2). The main effect of trial type was significant in the reaction time and accuracy rate [*F*(1, 49) = 356.48, *p* < 0.001, *η*^2^ = 0.88; *F*(1, 49) = 13.81, *p* < 0.005, *η*^2^ = 0.02], with a longer reaction time and lower accuracy rate for incongruent trials than for congruent trials (588 ± 77 ms vs. 491 ± 70 ms, 95.47% ± 6.39% vs. 99.02% ± 2.67%).

### ERP results

#### N2 component

**Figure 3:**
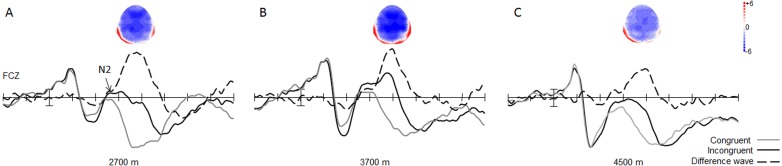
Grand average of ERP and topographical maps of N2. (A) The grand average of ERP elicited in the congruent (grey line), and the incongruent (black line) conditions, and the N2 difference wave (dash line) with its topographical map at FCz site for the 2,700 m group. (B) For the 3,700 m group. (C) For the 4,500 m group.

With regard to the amplitude of the N2 component, the main effect of trial type was significant [0.19 ± 0.57 µV vs. 3.58 ± 0.64 µV; *F*(1, 49) = 133.07, *p* < 0.001, *η*^2^ = 0.73], with respect to the amplitude of the N2 component, with larger N2 amplitudes in the incongruent trials, than in the congruent trials for all groups (Fig. 3). The interaction between trial type and group was significant [*F*(2, 49) = 3.37, *p* < 0.05, *η*^2^ = 0.12]. Simple effect analysis showed that the N2 wave was marginally significantly different between the 3,700-m and 4,500-m groups, under incongruent trial condition, with a smaller N2 amplitude in the 4,500-m group than in the 3,700-m group (1.27 ± 1.03 µV vs. −1.36 ± 0.96 µV, *F*(1, 2) = 1.98, *p* = 0.06). Moreover, post-hoc tests showed that the group difference between the 2,700-m and 3,700-m groups, and between the 2,700-m and 4,500-m groups were not statistically significant (*p* = 0.15, *p* = 0.68).

The main effect of group was significant [*F*(1, 2) = 3.37, *p* < 0.05, *η*^2^ = 0.12] for the N2 difference wave. Simple effect analysis showed that the N2 difference wave was more pronounced in the 2,700-m group than in the 4,500-m group, and more pronounced in the 3,700-m group than in the 4,500-m group (*p* < 0.05, −7.24 ± 0.75 µV for 2,700-m group; −7.16 ± 0.73 µV for 3,700-m group; and −4.95 ± 0.75 µV for the 4,500-m group). Moreover, post-hoc tests showed that the group difference between the 2,700-m and 3,700-m group was not statistically significant (Fig. 3). No other main effects or interactions were significant.

#### P3 component

The mean amplitude and the 50% area latency were used for analysis of the P3 component. The main effect of the trial type was statistically significant in terms of P3 latency, with a longer P3 latency for incongruent, than for congruent stimuli [494.22 ± 33.86 ms vs. 426.42 ± 32.07 ms; *F*(1, 49) = 216.34, *p* < 0.001, *η*^2^ = 0.82]. No other main effects or interactions were statistically significant.

## Discussion

Our study compared the neurocognitive features of conflict control in healthy young Tibetans living below and above 4,000 m using a flanker task, with an aim to identify whether 4,000 m was the conflict control impairment threshold. A conflict control effect was successfully elicited in the flanker task, as reflected by the behavioral and ERP results, with larger N2 amplitude in the incongruent trials which reflected the processing of conflict detection (Rueda et al., 2004). Moreover, longer P3 latency in the incongruent trials was due to increased stimulus evaluation or categorization time needed to determine the correct response in the parietal cortex (Rueda et al., 2004). The 4,000 m conflict control impairment threshold was established, as reflected by a smaller N2 difference wave in the group residing at an altitude above 4,000 m than in the groups residing below 4,000 m (3,700 and 2,700 m).

The principal finding of this study was that the conflict-monitoring stage of conflict control processing, as reflected by the N2 component, was significantly affected by various high altitudes. The N2 difference wave was calculated, to exclude the influence of congruent conditions, and to show the group difference in conflict control with clarity. Smaller N2 different wave was found in the 4,500-m group, compared to the 2,700-m group, similar to the group difference between the 3,700-m and 4,500-m groups. Moreover, the group difference between the 2,700-m group and 3,700-m group was not statistically significant. The N2 component was the indicator of conflict detection, the amplitude of N2 component is smaller in groups of individuals with impaired inhibitory functioning (Bokura, Yamaguchi & Kobayashi, 2005; Porjesz et al., 1987; Rentrop et al., 2011). The decreased amplitude of N2 different wave in the 4,500-m group may suggest that the conflict motoring processing in the flanker task was affected by 4,500 m altitude. According to earlier studies (Ma et al., 2015a; Ma et al., 2015b), inhibition processing was influenced by high-altitude exposure (3,700 m). Although the N2 amplitudes in the 2,700-m group and 3,700-m group were larger than the 4,500-m group, we could not conclude that the conflict-monitoring processing in these two altitude-groups were not affected by high altitude, due to the absence of a low-altitude control group. The results of our study may suggest that compared with that of 2,700 m and 3,700 m, the influence of 4,500 m of altitude on conflict-monitoring was more prominent. According to earlier studies (Dykiert et al., 2010; Virués-Ortega et al., 2011), 4,000 m maybe the conflict control impairment threshold. Cerebral auto-regulation may be lost at the transitional zone of approximately 4,000 m (Jansen et al., 2007). In our study, cognitive impairment was more obvious in the 4,500-m group and the difference between the 2,700-m group and 3,700-m group was not significant. Thus, these results may suggest that the adaptation to high-altitude environments may occur under 4,000 m for a population indigenous to high-altitudes. In other words, cognitive impairment may occur at the threshold of 4,000 m, at which the cerebral circulation fails to adequately compensate for hypoxia. No group difference was found under congruent trial conditions, similar to our earlier studies (Ma et al., 2015a; Ma et al., 2015a; Ma et al., 2015b). The result suggests that the impact of high altitude is limited to conflict-related cognitive processing, not general cognitive processing.

We did not find incongruent-P3 amplitude differences among the three high-altitude groups, contrary to our hypothesis. This may suggest that the conflict-resolving stage of the conflict control process in the flanker task did not differ among individuals living at these three high altitudes. As N2 and P3 represent separable cognitive processing in the flanker task, the incongruent-P3 amplitude was related to the late stage of the conflict control process (Ma et al., 2015b). The unaffected late processing was also reflected in the behavioral results with no significant group differences in the behavior results.

The differences in behavior were also not statistically significant across the three groups. This may be because Tibetan individuals are highly adapted to a high-altitude environment, with respect to behavior, irrespective of the altitude at which they are living. The lower sensitivity of the behavioral measure is probably another reason for the lack of significance in terms of behavior; EEG is more sensitive in measuring cognitive processing. Moreover, the flanker task may have been too easy for the subjects; based on the findings of our earlier studies, the group differences in behavioral results may become more prominent with tasks of greater difficulty (Ma et al., 2015a; Wang et al., 2014).

A limitation of our study is that although, the 4,000-m threshold was obvious in the conflict monitoring process in our study, the results of our study may not be generalizable to all individuals residing at other areas of high altitude around the world, because social class, parental education, and the presence of behavioral problems also affect neuropsychological functioning (Davis-Kean, 2005). Moreover, different ethnic groups show differences in physical function and adaptation to high-altitude hypoxia (Beall, 2000; Beall, 2007); thus, they may also have different cognitive impairment thresholds.

## Conclusions

In conclusion, our study demonstrates that the brains of Tibetans are influenced by hypoxia (Wilson, Newman & Imray, 2009), despite physical adaptation by long-term natural selection in these individuals, with evolutionary genetic and physical structural adaptations (Beall, 2007). High altitude alters conflict control processing at the conflict-monitoring stage, as evidenced by the incongruent-smaller N2 amplitude in the 4,500-m group. Moreover, there may be a cognitive impairment threshold at around 4,000 m, as the influence of extreme high altitude on conflict control was more marked, above this level.

##  Supplemental Information

10.7717/peerj.7269/supp-1 Data S1 Raw data exported from the neuroscan applied for data analyses and preparation for Figs. 2 and 3

## Additional Information and Declarations

### Data Availability

The following information was supplied regarding data availability: The raw data are available at Figshare: wang (2019): flanker cnt data. figshare. Dataset. https://doi.org/10.6084/m9.figshare.7789616.v1. wang (2019): flanker behavior.zip. figshare. Dataset. https://doi.org/10.6084/m9.figshare.7789574.v1.
